# A noble bimetal oxysulfide Cu*V*OS catalyst for highly efficient catalytic reduction of 4-nitrophenol and organic dyes

**DOI:** 10.1039/c9ra05172d

**Published:** 2019-10-07

**Authors:** Huizhi Sun, Osman Ahmed Zelekew, Xiaoyun Chen, Yuanbo Guo, Dong-Hau Kuo, Qingxin Lu, Jinguo Lin

**Affiliations:** College of Materials Engineering, Fujian Agriculture & Forestry University Fuzhou 350002 China fjchenxy@126.com fjlinjg@126.com; Department of Materials Science and Engineering, Adama Science and Technology University Adama Ethiopia osman.ahmed@astu.edu.et; Department of Materials Science and Engineering, National Taiwan University of Science and Technology No. 43, Sec. 4, Keelung Road Taipei 10607 Taiwan dhkuo@mail.ntust.edu.tw

## Abstract

A novel copper–vanadium bimetallic oxysulfide (Cu*V*OS) nanoparticle catalyst was successfully synthesized by a facile method. The samples were characterized by X-ray photoelectron spectrometry (XPS), X-ray diffractometry (XRD), field-emission scanning electron microscopy (FE-SEM), UV-Vis diffuse spectroscopy (DRS), Fourier transform infrared spectroscopy (FTIR), and N_2_ adsorption–desorption isotherms. In order to check the catalytic efficiencies toward reduction reaction, 4-nitrophenol (4-NP) and other organic dyes such as rhodamine-B (RhB), methylene blue (MB), and methyl orange (MO) were used. The results showed that the Cu*V*OS prepared in the presence of a suitable amount of N_2_H_4_ during the synthesis of the nanoparticles exhibited the fastest reduction capabilities by using NaBH_4_ as a reducing agent. It was demonstrated that a 100 mL 4-NP (20 ppm) solution was completely reduced by 5 mg Cu*V*OS-3 within 2 min. Moreover, the complete reduction of 100 mL of MO, RhB, and MB solutions of 100 ppm was also achieved by 5 mg Cu*V*OS-3 within 2 min, 6 min, and 5 min, respectively. Hence, the Cu*V*OS is an efficient catalyst for reducing 4-NP and organic dyes and can have great potential for industrial application.

## Abstract 2

A novel Cu*V*OS catalyst was successfully synthesized by a facile method. The Cu*V*OS with optimum amount of N_2_H_4_ had higher catalytic activity.

## Graphical abstract

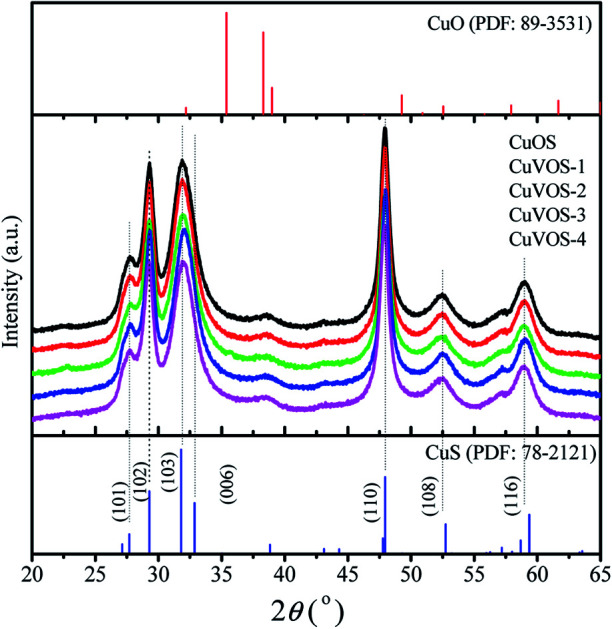

## 1. Introduction

Recently, the rapid growth of population and industry became the major concern regarding environmental and energy issues.^1^ In particular, the discharge of toxic nitroaromatic organic compounds used in the manufacturing of pharmaceuticals, pigments, explosives, plastics, pesticides and fungicidal agents is potentially dangerous to humans and the environment.^2–4^ Among the nitroaromatic compounds, 4-nitrophenol (4-NP) and its derivatives are the most toxic and can be the cause for the damage of the central nervous system, kidney, liver and human blood.^5,6^ In addition to nitroaromatic compounds, synthetic dyes discharged from different industries such as paper and pulp manufacturing, leather industries, cloth dyeing, and plastics coloring have been used extensively and are also a source of environmental pollution.^6–8^ Particularly, strong color and pigment dyes discharged into the aquatic system cause serious environmental problems and are also the major concern for water pollution.^9–12^ Hence the reduction and removal of toxic organic compounds from aquatic environments is a crucial mission.

In the recent years, many researchers have tried to remove toxic organic compounds and also decolorize wastewater by using different techniques. Among the methods used, photocatalytic degradation, adsorption, membrane flocculation, filtration, and reduction technological applications are reported by different scholars.^13–19^ Sun *et al.* used a carbon microsphere-loaded copper-based catalyst for the degradation of organic dyes in aqueous solution.^20^ Liu *et al.* also studied the synthesis of various NiCo_2_O_4_ structures to degrade organic dyes under microwave catalysis.^21^ Zhao *et al.* studied the photocatalytic degradation of 4-nitrophenol in the presence of H_2_O_2_ in a UV-irradiated water dispersion system using TiO_2_ catalyst doped with different amounts of iron, and systematically explored 1% Fe–TiO_2_ catalyst.^22^ Moreover, the catalytic reduction of 4-nitrophenol was also conducted by Zelekew *et al.* with silica-supported metal oxide-based catalysts.^23,24^ Elfiad *et al.* also used hematite to explore the conversion of nitrophenol (4-NP) to 4-aminophenols (4-AP).^25^ Ajmal *et al.* also reported the simultaneous catalytic degradation/reduction of multiple toxic organic compounds by modifiable p(methacrylic acid-*co*-acrylonitrile)–M (M: Cu, Co) microgel catalyst composites.^26^ The reduction of rhodamine B by using magnetically recyclable Ag–Fe_3_O_4_ composite catalyst haven also reported by Ai *et al.*^27^ However, the synthesis of the catalyst at low temperature process with efficient and low-cost catalyst for wastewater treatment is still the biggest challenge. Therefore, the development of straight forward methods for preparing efficient catalysts at low temperature is mandatory. Moreover, the existence of bimetallic compound catalyst is expected to enhance the catalytic activities by the synergistic effect.

In our previous work, the synthesis of the Zn(O,S) oxysulfide has been used for hydrogen production.^28^ The copper-based oxysulfide bimetallic CuNiOS catalysts with the effect of the Cu^+^/Cu^2+^ ratio for chromium reduction was also reported by our group. However, there was no report on Cu*V*OS bimetallic catalysts for the removal or reduction of organic compounds. This study aims to synthesize a copper-based bimetallic oxysulfide Cu*V*OS catalyst for the catalytic reduction of toxic organic compounds. The catalytic performance of the catalyst is checked by the reduction of 4-NP and other organic dyes such as MB, MO, and RhB.

## 2. Experimental

### 2.1 Synthesis of Cu*V*OS

In a particular procedure, 4.8 g of copper nitrate [Cu(NO_3_)_2_·2.5H_2_O] and 1.0 g of ammonium metavanadate (NH_4_VO_3_) were dissolved into 900 mL DI water under vigorously stirring. Then, 100 mL 0.3 mol L^−1^ thioacetamide (CH_3_CSNH_2_, TAA) was added into the solution drop by drop. After 30 minutes stirring at room temperature, the mixture was then heated to 90 °C and then 0.4 mL of hydrazine hydrate (N_2_H_4_) was slowly dropped into the mixture followed with 2 h reaction. To optimize the amount of the added hydrazine, 0, 0.2, 0.4, and 0.6 mL of hydrazine were added to form products labeled as Cu*V*OS-1, Cu*V*OS-2, Cu*V*OS-3, and Cu*V*OS-4, respectively. Subsequently, the obtained precipitate was washed, centrifuged, and dried with rotary evaporation to obtain Cu*V*OS catalysts prepared with different N_2_H_4_ contents. For comparison purpose, CuOS was prepared with the same procedure without the addition of vanadium source.

### 2.2 Characterizations

The XRD pattern of the CuOS and Cu*V*OS catalysts was characterized by the Rigaku X-ray diffractometer with the Cu Kα radiation (*λ* = 1.5406 Å) sources. The XPS analysis was performed by the PHI5700 photoelectron spectrometer with Al Kα X-ray (*hν* = 1486.6 eV) radiation, and C 1s at 284.62 eV was used for calibration purpose. The FTIR analysis was performed by Agilent Digilab FTS-3500 Fourier transforms infrared spectrometer. The surface morphologies of the samples were checked by FE-SEM (HITACHI SU-8010 microscope) with an accelerating voltage of 10 kV and by TEM and HR-TEM (Tecnai F20 G2 microscope). The UV-Vis DRS analysis was characterized by TU-1901 UV-Vis spectrophotometer equipped with an integrating sphere, and BaSO_4_ was as a reference.

### 2.3 Catalytic reduction experiments

#### 2.3.1 Catalytic reduction of 4-NP

In the particular procedure, 5 mg Cu*V*OS catalyst was added into 100 mL (20 mg L^−1^) 4-NP aqueous solution. Then, freshly prepared, 3 mL (0.2 mol L^−1^) of NaBH_4_ aqueous solution was added to the mixture. Subsequently, 2 mL sample was taken from the reactor at a regular interval of time. The reduction progress of the resulting 4-NP was monitored by TU-1901 UV-Vis spectrophotometer at room temperature. The Lambert–Beer law was used to calculate their concentration.

In the case of organic dye reduction, MO, MB, and RhB dyes were selected to test the catalytic performance of the samples. In a particular procedure, 5 mg Cu*V*OS catalyst was added into 100 mL (100 ppm) MO solution. Then, 3 mL 0.2 mol L^−1^ NaBH_4_ solution was added to the above solution. Subsequently, 2 mL of sample was removed from the reactor at a regular interval time. For MB and RhB dyes, the concentration of the dye and the amount of NaBH_4_ used were similar with the reduction of MO dye mentioned above. According to the absorbance maxima of MO, MB, and RhB at 465 nm, 663 nm, and 554 nm, respectively, the retained concentration was calculated with the Lambert–Beer law.

To evaluate the stability of the catalyst, the Cu*V*OS-3 catalyst was taken and used repeatedly. In a particular procedure, 200 mg of Cu*V*OS-3 catalyst was added into 4000 mL (20 mg L^−1^) 4-NP aqueous solution in to 5000 mL beaker. Then, freshly prepared, 120 mL (0.2 mol L^−1^) of NaBH_4_ aqueous solution was added in to the mixture and reduction progress of the resulting 4-NP was then monitored by TU-1901 UV-Vis spectrophotometer. After reaction, the solution was settled by gravity and the upper layer solution was poured out. The remaining solution was separated with centrifuged and the catalyst was used for the next reactions. After five time runs, the catalyst was collected and dried with oven at 60 °C for 2 h and used for XPS and XRD analysis.

## 3. Results and discussion

### 3.1 XRD analysis

**Fig. 1:**
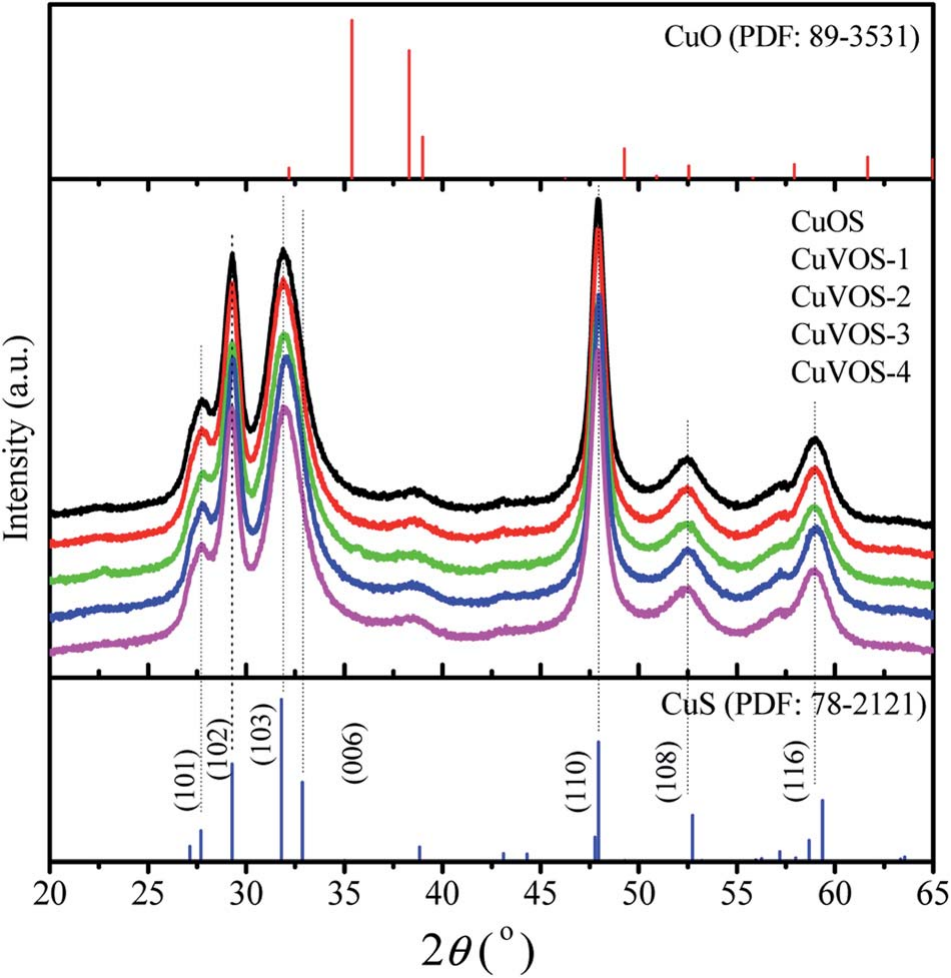
XRD diffraction patterns of Cu*V*OS catalysts with different amounts of hydrazine.

Fig. 1 shows the XRD diffraction patterns of Cu*V*OS. The diffraction peaks positions of Cu*V*OS were correspondent with the structure of hexagonal CuS covellite (PDF #89-2531). The major peaks of the samples were located at 2*θ* value of 27.66°, 28.36°, 29.62°, 31.81°, 32.72°, 48.23°, and 52.75°, which were attributed to (100), (101), (102), (103), (006), (110), and (108) crystal planes, respectively. As we have seen from the XRD peaks, there was a little shift to lower angle as we compared from CuS standard peaks. Moreover, the XRD diffraction patterns had no second phases of CuO, Cu_2_O, Cu_2_S, V_2_O_5_, and V_2_S_5_, which indicated that the Cu*V*OS catalyst is a solid solution. The peak full width at half maxima indicated that a little effect of adding different amount of N_2_H_4_ was observed on the crystal structure and crystallinity. The average crystal sizes of Cu*V*OS catalysts prepared with different amounts of N_2_H_4_ were also calculated by Scherrer formula and showed in Table 1. As it is observed from Table 1, the average the crystalline size for catalyst was 12.7–13.5 nm.

**Table 1:** XPS composition analyses of Cu*V*OS catalysts

Catalyst	Molar percentage (%)				Cu molar percentage (%)		Cu^+^/Cu^2+^ molar ratio	O molar percentage (%)		S molar percentage (%)		S^2−^/S^6+^ molar ratio	Crystal sizes (nm)
	Cu	V	O	S	Cu(i)	Cu(ii)		O–H	O_lattice_	S^2−^	S^6+^		
Cu*V*OS-1	35.65	2.69	21.03	40.67	65.49	34.51	1.90	29.96	70.04	88.93	11.07	8.03	12.7
Cu*V*OS-2	35.37	2.71	21.27	40.65	69.33	30.67	2.26	30.84	69.16	87.22	12.78	6.82	13.2
Cu*V*OS-3	35.26	2.72	21.15	40.87	72.02	27.98	2.57	30.73	69.27	85.65	14.35	5.97	13.5
Cu*V*OS-4	35.62	2.68	20.98	40.72	75.31	24.69	3.05	30.50	69.50	84.16	15.84	5.31	13.1
CuS commercial	48.91	0	2.41	48.68	0	100	—	—	—	—	—	—	—
Cu*V*OS-3 after reaction	35.40	2.65	21.07	40.88	72.05	27.95	2.58	30.55	69.45	85.47	14.53	5.88	—

### 3.2 XPS analysis

**Fig. 2:**
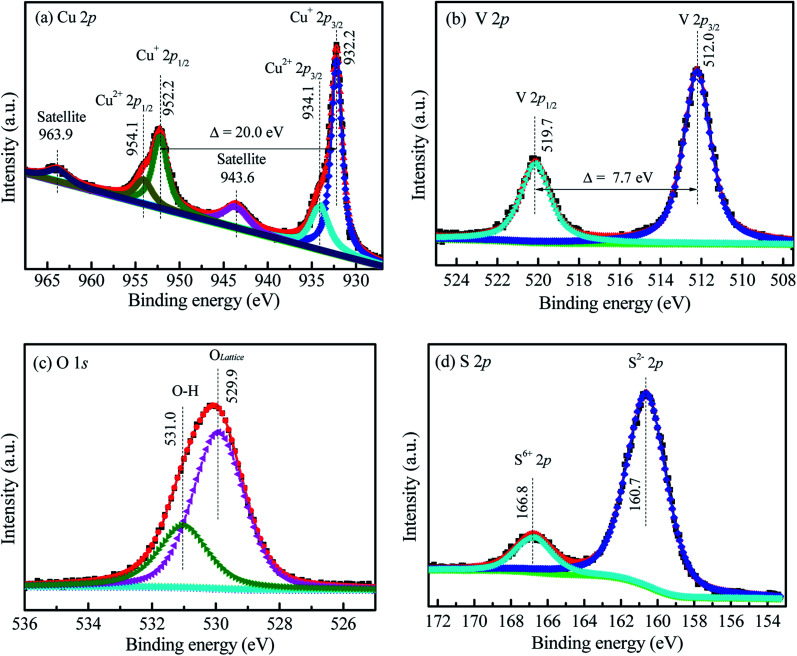
High resolution (a) Cu 2p, (b) V 2p, (c) O 1s, and (d) S 2p XPS spectra of Cu*V*OS-3.

Fig. 2a also shows the Cu 2p XPS spectra of Cu*V*OS-3. The peaks located at 932.22 eV and 952.22 eV represents for the Cu 2p_3/2_ and Cu 2p_1/2_, respectively, which indicates the presence of Cu(i) oxidation state in the sample.^29^ Moreover, the peaks for Cu 2p_3/2_ and Cu 2p_1/2_ located at 934.10 eV and 954.10 eV, respectively, indicates the presence of Cu(ii) oxidation state.^30,31^ The satellite peaks are also located at 943.7 eV and 963.1 eV. The results indicated that the copper is existed in the forms of Cu(i) and Cu(ii) oxidation states in the sample.^30,31^ Moreover, Fig. 2b also shows the V 2p XPS spectra in the Cu*V*OS-3 catalysts. The peaks of V 2p_3/2_ and V 2p_1/2_ located at 512.0 eV and 519.7 eV, respectively, which indicates the existence of V(iv) oxidation state in the Cu*V*OS-3 sample.^32^Fig. 2c shows the O 1s XPS spectra in the Cu*V*OS-3 catalyst. The peaks at 531.0 eV and 529.9 eV attributed to the hydroxyl oxygen and the lattice oxygen, respectively.^33^ The S 2p XPS spectra in the Cu*V*OS-3 catalyst also showed in the Fig. 2d. The S 2p peaks at 160.7 eV indicates the formation of S^2−^ and the peak at 166.8 eV also showed the existence of S^6+^ in the Cu*V*OS-3 catalyst.^34^ Moreover, the chemical compositions of the Cu*V*OS with different amount of hydrazine, and the Cu*V*OS-3 catalyst after reused are shown on Table 1 according to the peak-fitting area. As it is indicated from Table 1, the molar ratios of Cu^+^/Cu^2+^ were increased as the amount of hydrazine increases due to the reduction of Cu^2+^ to Cu^+^. The O_lattice_ composition was almost constant with increasing hydrazine. However, the molar ratio of the S^2−/^S^6+^ decreases with increasing the amount of hydrazine.

### 3.3 FE-SEM and TEM analyses

**Fig. 3:**
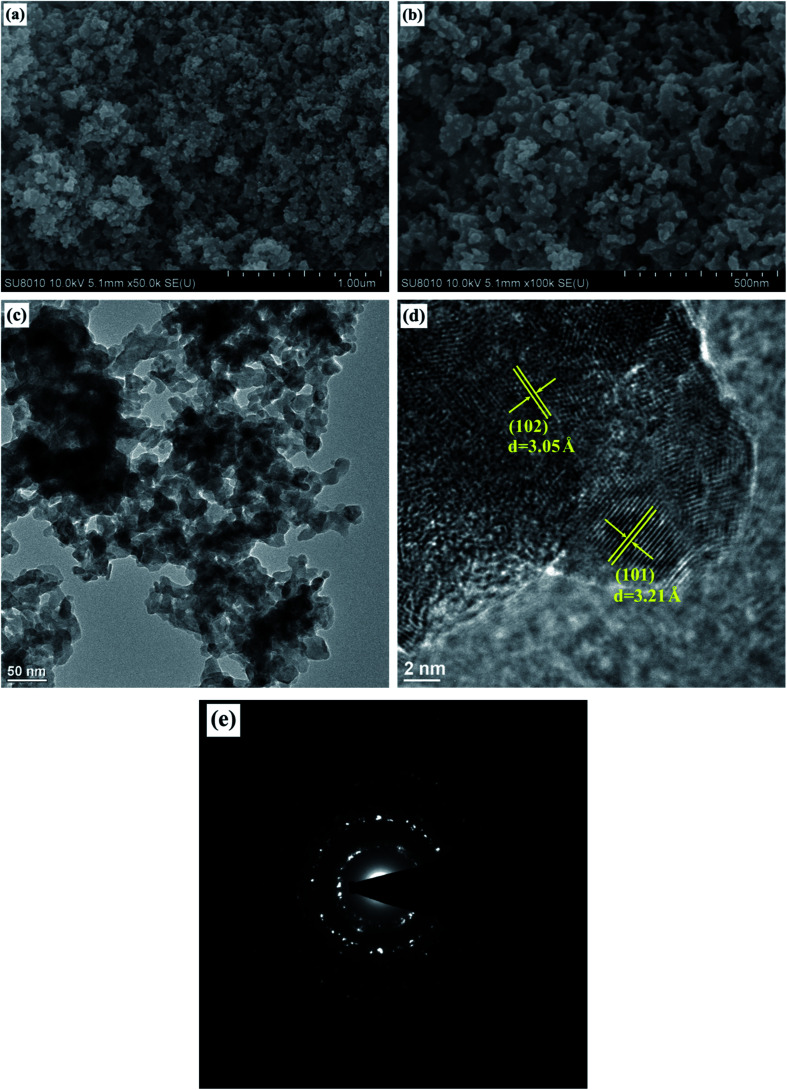
(a and b) FE-SEM, (c) TEM, and (d) HR-TEM images, and (e) SAED pattern of Cu*V*OS-3 catalyst.

Fig. 3a and b show the FE-SEM images of Cu*V*OS-3 catalyst. As we checked the SEM images, the Cu*V*OS nanoparticle and agglomeration with 50–200 nm were observed. Fig. 3c also indicates the TEM image of Cu*V*OS-3 and it also further confirms the nanoparticle agglomeration. Fig. 3d displays the HR-TEM image of Cu*V*OS-3 and the *d*-space values of 3.21 Å and 3.05 Å in the image were attributed to hexagonal CuS (101) and (102) planes, respectively. Fig. 3e also shows the selected area electron diffraction (SAED) pattern of Cu*V*OS-3. The ring patterns illustrate its polycrystalline nature. In general, the XRD, XPS, and TEM analysis further confirms the formation of the major phase of CuS in the Cu*V*OS solid solution.

### 3.4 BET and pore size analysis

**Fig. 4:**
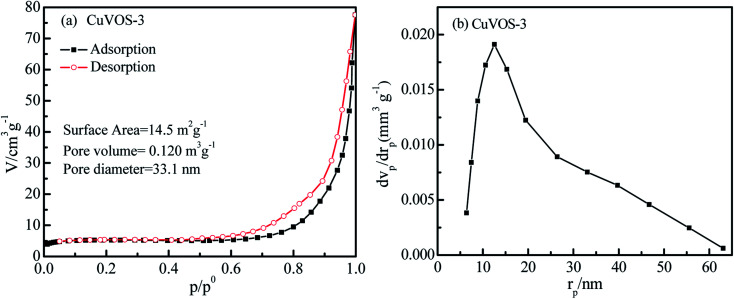
(a) Nitrogen adsorption–desorption isotherm and (b) the pore size distribution curve of Cu*V*OS-3.

Fig. 4a displays the N_2_ adsorption–desorption isotherm of Cu*V*OS-3. As we can see from the Fig. 4a, it has a consistent trend with the type IV isotherm of the hysteresis loop for a mesoporous characteristics when the relative pressure (*P*/*P*_0_) is 0.75–1.0.^35^ Moreover, the pore size distribution curve of the samples is showed in Fig. 4b. The surface area (*S*_BET_), total pore volume, and average pore diameter of Cu*V*OS-3 were 14.5 m^2^ g^−1^, 0.120 m^3^ g^−1^, and 33.1 nm, respectively.

### 3.5 FT-IR analysis

**Fig. 5:**
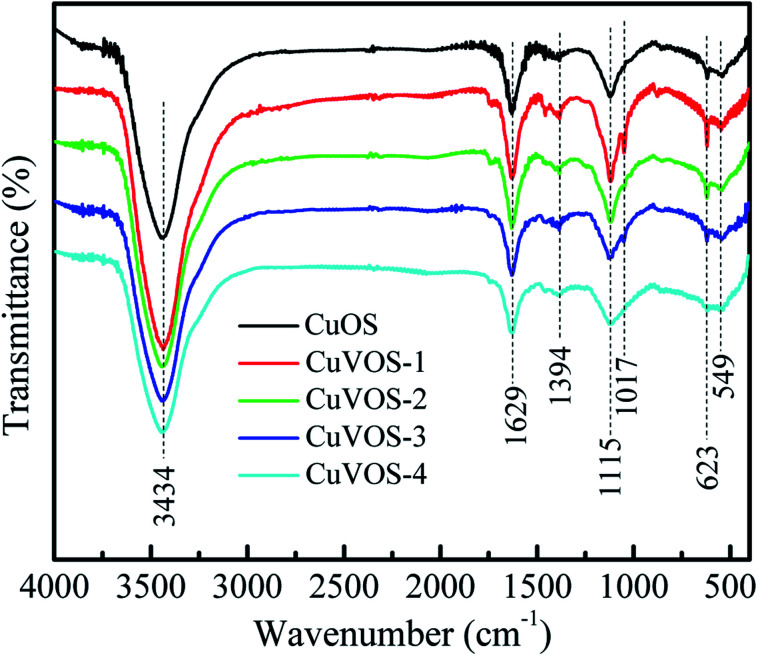
FTIR spectra of CuOS and Cu*V*OS prepared with different N_2_H_4_ contents.

Fig. 5 shows the FTIR spectra of CuOS and Cu*V*OS prepared in the presence of different amounts of N_2_H_4_. The peaks located at 3434 and 1629 cm^−1^ corresponds to the vibration mode of the stretched and bending vibration from surface adsorbed water or surface hydroxyl groups.^36,37^ The peak at 623 cm^−1^ corresponded to the Cu–O stretching vibration.^38,39^ The peak at 1115 cm^−1^ was attributed to the S–O stretching vibrations in Cu*V*OS.^40,41^ The existence of the peak around 549 cm^−1^ also indicated the presence of Cu–S stretching vibration.^42^

### 3.6 UV-Vis absorption spectra

**Fig. 6:**
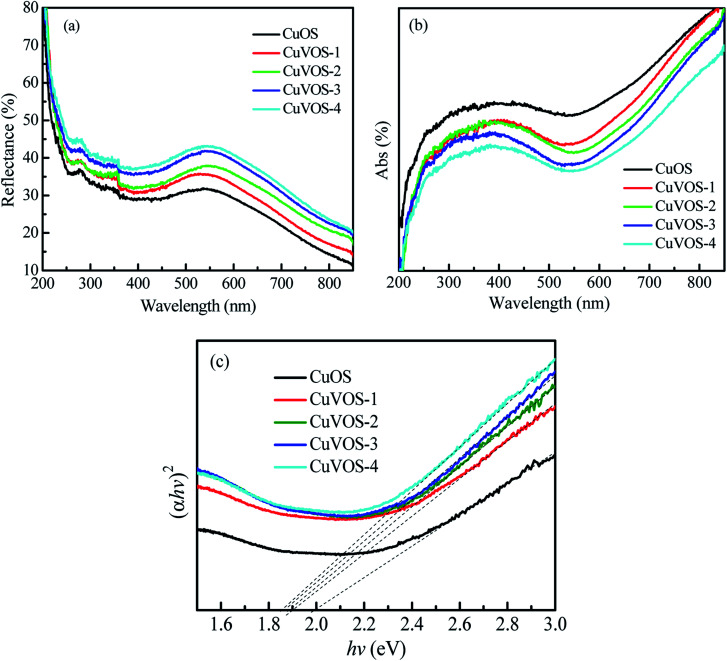
Optical properties of CuOS and Cu*V*OS series catalysts evaluating with (a) UV-Vis reflectance spectroscopy and (b) UV-Vis absorption spectroscopy. (c) The (*αhν*)^2^–*hν* plot from the UV-Vis absorption measurements.

Fig. 6a and b show the UV-Vis reflection and absorption spectroscopy of the CuOS and Cu*V*OS, respectively. From the UV-Vis absorption spectra, the direct band gap was calculated with the equation as follows: (*αhν*)^2^ = *k*(*hν* − *E*_g_).^43^Fig. 6c indicates the (*αhν*)^2^–*hν* curves of Cu*V*OS and CuOS catalysts. The *E*_g_ values were to be 1.6–1.8 eV for Cu*V*OS-1, 2, 3, 4, and ∼2.0 eV for CuOS. As it is compared from CuOS, the band gap of Cu*V*OS-1, 2, 3, 4 had smaller *E*_g_ values. The monocrystalline CuO of *E*_g_ = 1.2–1.4 eV, Cu_2_O of 2.0–2.2 eV, Cu_2_S of 1.2–1.25 eV, CuS of 2.15–2.36 eV are known. The band gaps of the samples further indicate that Cu*V*OS is a bimetal oxysulfide solid solution.

### 3.7 Catalytic reduction of 4-nitrophenol

**Fig. 7:**
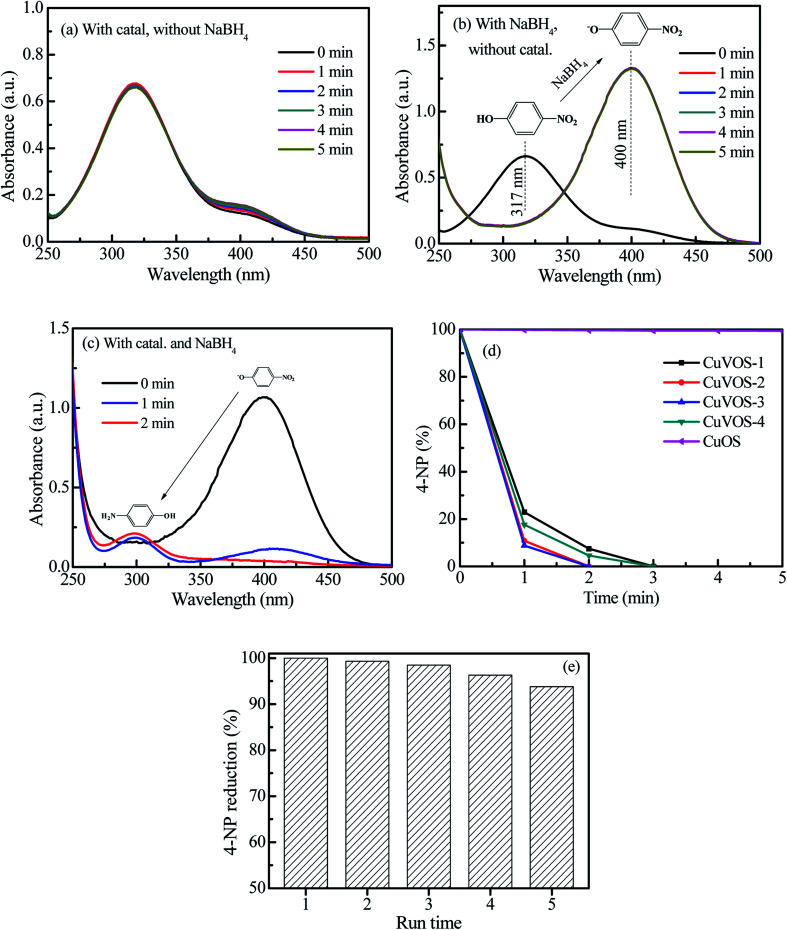
(a) Reduction of 4-NP with Cu*V*OS-3 catalyst without NaBH_4_, (b) reduction of 4-NP by NaBH_4_ without catalyst, (c) reduction of 4-NP by Cu*V*OS-3 catalyst with NaBH_4_, (d) reduction of 4-NP by Cu*V*OS catalyst prepared at different N_2_H_4_ contents with NaBH_4_, (e) the reusability of Cu*V*OS-3 for 4-NP reduction.

Fig. 7a and b show the control reduction of 4-NP by Cu*V*OS catalysts. As is it observed from the Fig. 7a, the 4-NP solution was not changed in the presence of Cu*V*OS catalyst. However, the addition of NaBH_4_ in the 4-NP solutions changed the absorption peak of the 4-NP from 317 nm to 400 nm (Fig. 7b). The shift of the absorption peak in the presence of NaBH_4_ reagent indicates the formation of 4-NP ion, which is also accompanied by a light yellow to deep yellow color change.^24^ Moreover, Fig. 7c shows the reduction reaction of 4-NP solution with the Cu*V*OS-3 catalyst in the presence of NaBH_4_. It is indicated that the absorption peak at 400 nm rapidly decreased and subsequently a new peaks appeared at 300 nm which belonged to 4-AP.^6,24^Fig. 7d shows the percentage of the 4-NP reduction reaction over the CuOS and Cu*V*OS prepared with different amounts of N_2_H_4_. As indicated from the Fig. 7d, the reduction of 4-NP with Cu*V*OS in the presence of NaBH_4_ was faster. However, the reduction of 4-NP was insignificant in the presence of CuOS. The kinetic rates of reaction with Cu*V*OS catalyst prepared with different amounts of N_2_H_4_ followed the order: Cu*V*OS-3 > Cu*V*OS-2 > Cu*V*OS-4 > Cu*V*OS-1 > CuOS. This indicated that the Cu*V*OS prepared with suitable amount of N_2_H_4_ will have an appropriate *n*[Cu(i)/Cu(ii)] molar ratio and is expected to generate the anion vacancy due to the high Cu^+^ in Cu^2+^S^2−^ covellite structure. Moreover, the addition of N_2_H_4_ also leads to the change the surface defect state of Cu*V*OS in order to facilitate its interaction with 4-NP. The Cu*V*OS with the suitable *n*[Cu(i)/Cu(ii)] molar ratio can also transfer the electron between the Cu(i) and Cu(ii) species.^8,44^

Moreover, the catalytic performances of our catalysts were also compared with other literature reports. As it is indicated in Table 2, complete reduction of 4-NP was achieved by 5 mg Cu*V*OS-3 catalyst in the presence of NaBH_4_ within 2 min reaction time. Therefore, our catalysts prepared with facile method had comparable catalytic performance towards reduction of 4-NP with reported literatures. Table 2 shows the comparison of the catalytic activity of various catalysts reported in literature with Cu*V*OS.

**Table 2:** Comparison of the catalytic activity of various catalysts reported in literature for the reduction of 4-NP by NaBH_4_

No.	Catalysts/amount	Time (s)	Kinetic rate constant, *k*_app_ (s^−1^)	Ratio constant, *K* (s^−1^ g^−1^)	Ref.
1	Co_3_O_4_/100 mg	120	0.013	0.13	45
2	NiPt-0.6%/15.5 mg	140	0.01882	1.2	46
3	CuO/100 mg	40	0.019	0.19	45
4	Cu_2_O–Cu–CuO/1 mg	180	0.0156	15.6	47
5	SiO_2_@Cu_*x*_O@TiO_2_/10 mg	210	0.025	2.5	24
6	CuOS/5 mg	300	—	—	This work
7	Cu*V*OS-1/5 mg	180	0.022	4.4	This work
8	Cu*V*OS-2/5 mg	120	0.037	7.4	This work
9	Cu*V*OS-3/5 mg	120	0.041	8.1	This work
10	Cu*V*OS-4/5 mg	180	0.027	5.4	This work

**Fig. 8:**
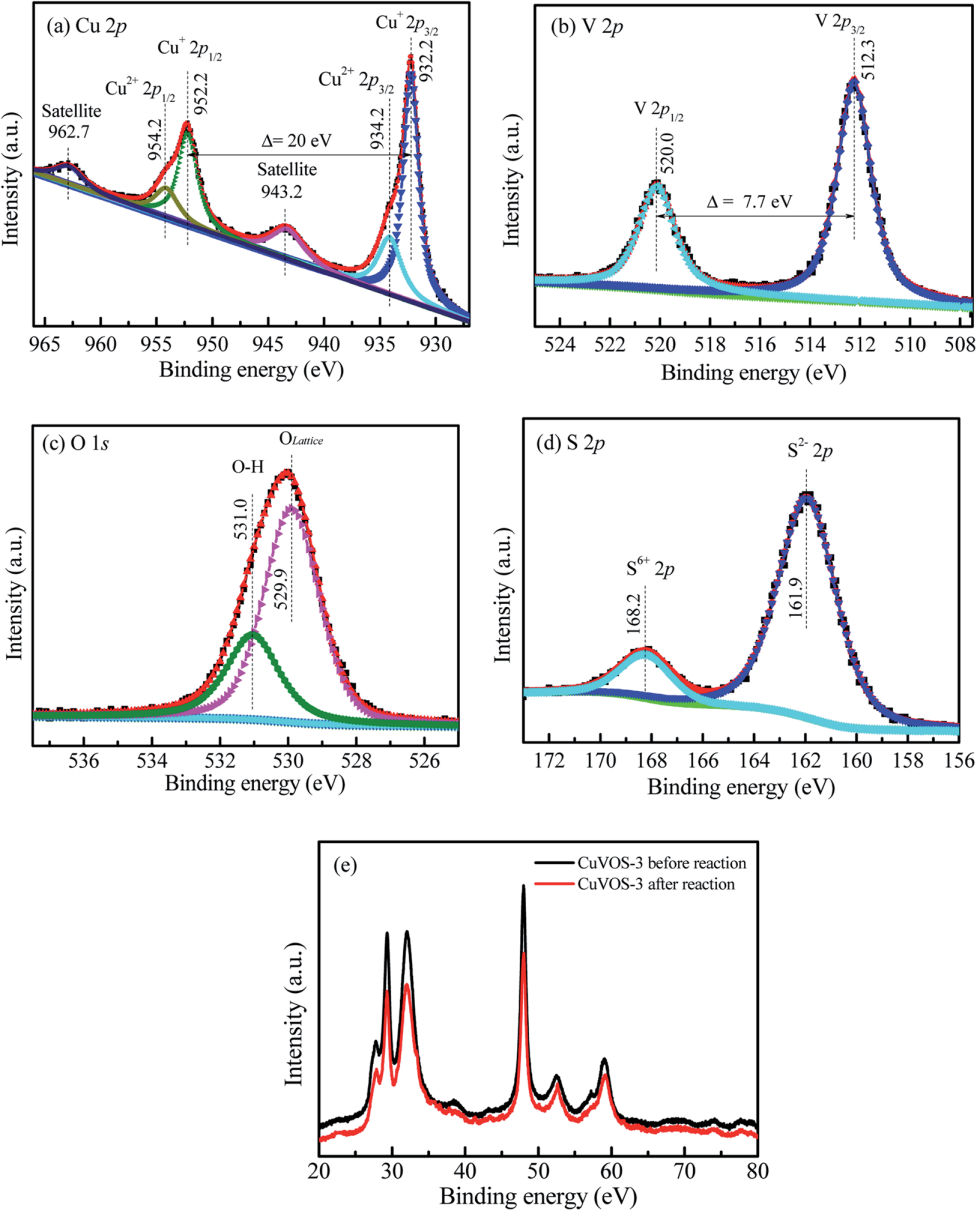
High resolution (a) Cu 2p, (b) V 2p, (c) O 1s, and (d) S 2p XPS spectra of Cu*V*OS-3 after reaction. (e) XRD diffraction patterns of Cu*V*OS-3 for before and after reaction.

Furthermore, the catalytic stability was also studied by using the Cu*V*OS-3 catalyst and its performance is shown in the Fig. 7e. After the 5^th^ run, the Cu*V*OS-3 had a very good reduction capability of 4-NP. In order to check the catalyst stability, the XPS analysis was performed after reduction of 4-NP. Fig. 8a shows the high resolution Cu 2p XPS spectrum of Cu*V*OS-3 after reduction reaction. The peaks located at 932.1 eV and 952.1 eV for Cu 2p_3/2_ and Cu 2p_1/2_, respectively, indicate the existence of Cu(i).^29^ The Cu(ii) oxidation state peaks of Cu 2p_3/2_ and Cu 2p_1/2_ were located at 934.1 eV and 954.1 eV, respectively.^30,31^ According to the quantitative analysis by integrating the peak area, the Cu(i)/Cu(ii) molar ratios of catalysts were calculated to be close to the Cu*V*OS-3 catalyst before reaction. Fig. 8b shows the high resolution V 2p XPS spectrum of Cu*V*OS-3 after reaction. The peaks of located at 512.0 eV and 519.7 eV were attributed to V 2p_3/2_ and V 2p_1/2_, respectively, which indicated the V(iv) oxidation state.^32,33^Fig. 8c shows the high resolution O 1s XPS spectrum of Cu*V*OS-3 after reaction. The peak located at 532.2 eV was contributed from hydroxyl oxygen, and at 530.1 eV from lattice oxygen. Fig. 8d shows the high resolution S 2p XPS spectrum of Cu*V*OS-3 after reaction. The S 2p peaks at 165.6 eV and 171.9 eV belonged to the S^2−^ and S^6+^, respectively.^34,48^ According to the quantitative analysis by integrating the peak area, the S^6+^/S^2−^ molar ratios of catalysts were calculated to be close to the Cu*V*OS-3 catalyst before reaction as it is shown in Table 1. Furthermore, Fig. 8e shows the XRD diffraction patterns of Cu*V*OS-3 before and after reaction. It can be seen that the peaks position of Cu*V*OS-3 before and after reaction are similar, but intensity of the peaks after reaction is little decreased. This result indicates that the catalyst is highly stable and can be used for multiple times.

### 3.8 Reduction activity on organic dyes

**Fig. 9:**
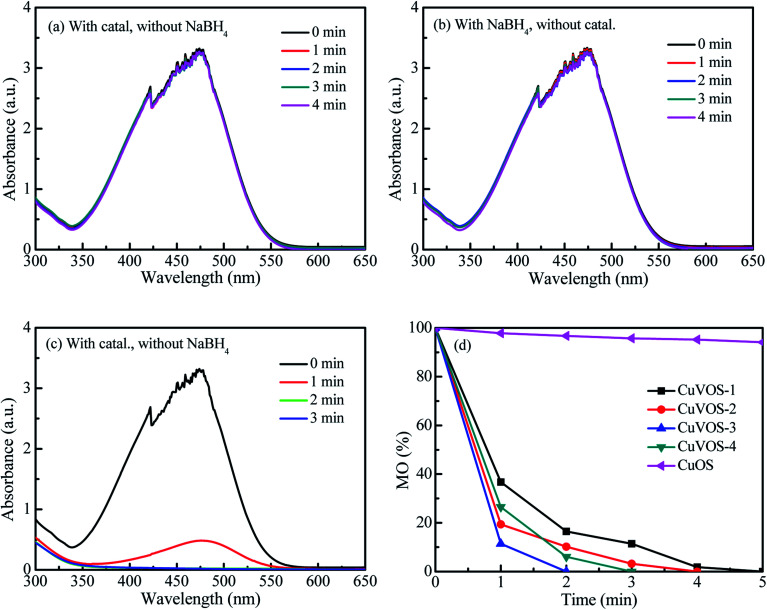
(a) Reduction of MO by Cu*V*OS-3 catalyst without NaBH_4_, (b) reduction of MO with NaBH_4_ without catalyst, (c) reduction of MO by Cu*V*OS-3 catalyst with different NaBH_4_, (d) Reduction of MO with Cu*V*OS series catalysts at different reaction times.

Fig. 9 shows the reduction of MO with Cu*V*OS catalyst. The control experiments were shown in Fig. 9a and b. As observed in Fig. 9a and b, there were no reduction reactions by using only catalyst or only NaBH_4_. However, the complete reduction of MO within 2 min was taken place in the presence of both Cu*V*OS-3 catalyst and NaBH_4_ (Fig. 9c). Moreover, Fig. 9d shows the pseudo first-order apparent reaction rate constant (*k*_app_) of MO reduction over Cu*V*OS catalysts having the order of reaction as follows: Cu*V*OS-3 (*k*_app_ = 1.94 min^−1^) > Cu*V*OS-4 (*k*_app_ = 1.40 min^−1^) > Cu*V*OS-2 (*k*_app_ = 1.07 min^−1^) > Cu*V*OS-1 (*k*_app_ = 0.70 min^−1^) > CuOS (*k*_app_ = 0.01 min^−1^). It is noted that the completed reduction of MO was achieved within as 2 minutes by Cu*V*OS-3 catalyst, while CuOS only achieved for 8% in 10 minutes.

**Fig. 10:**
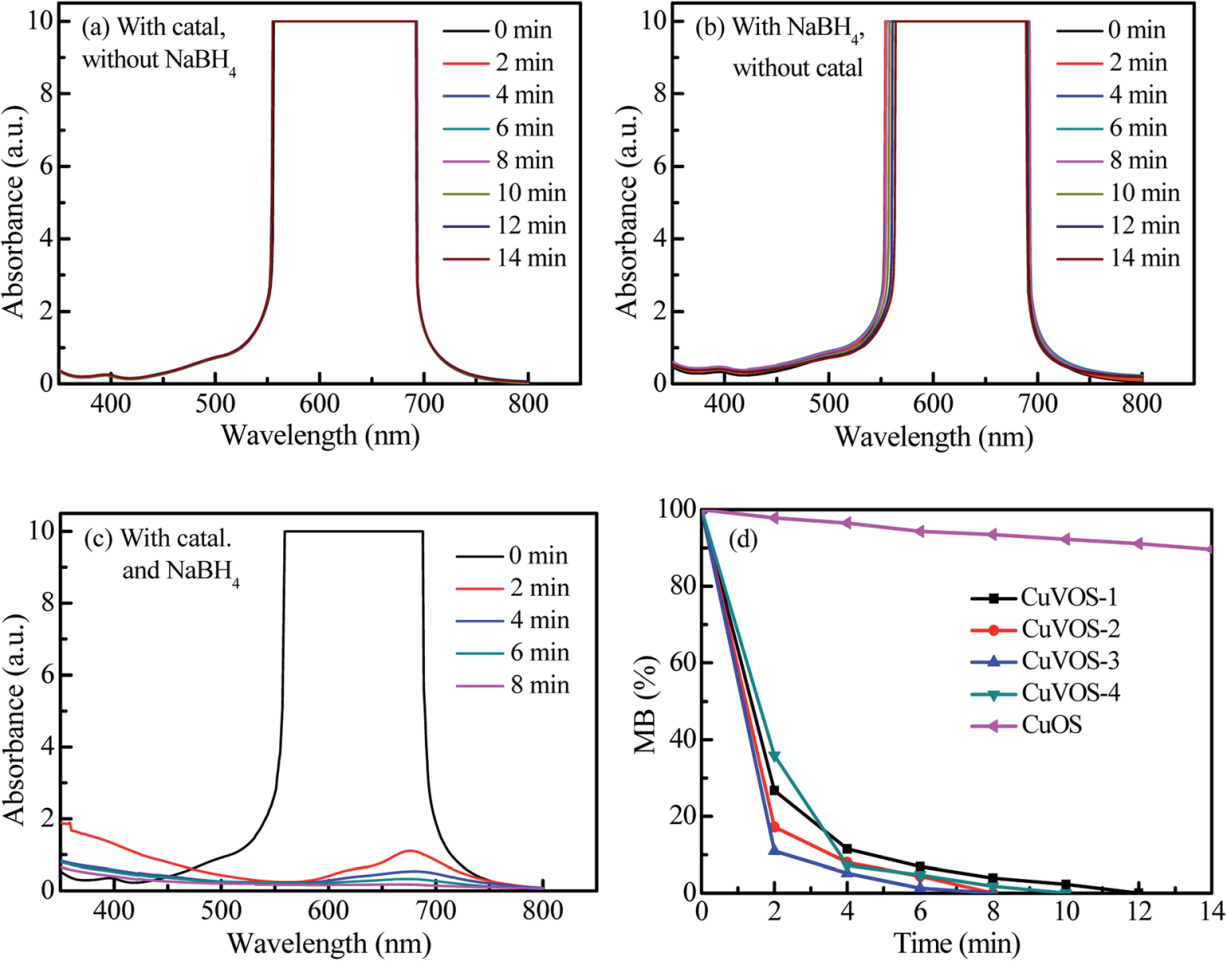
(a) Reduction of MB with Cu*V*OS-3 without NaBH_4_, (b) reduction of MB by NaBH_4_ without catalyst, (c) reduction of MB by Cu*V*OS-3 catalyst with NaBH_4_, and (d) reduction of MB by Cu*V*OS catalysts prepared at different N_2_H_4_ contents with NaBH_4_ under dark.

**Fig. 11:**
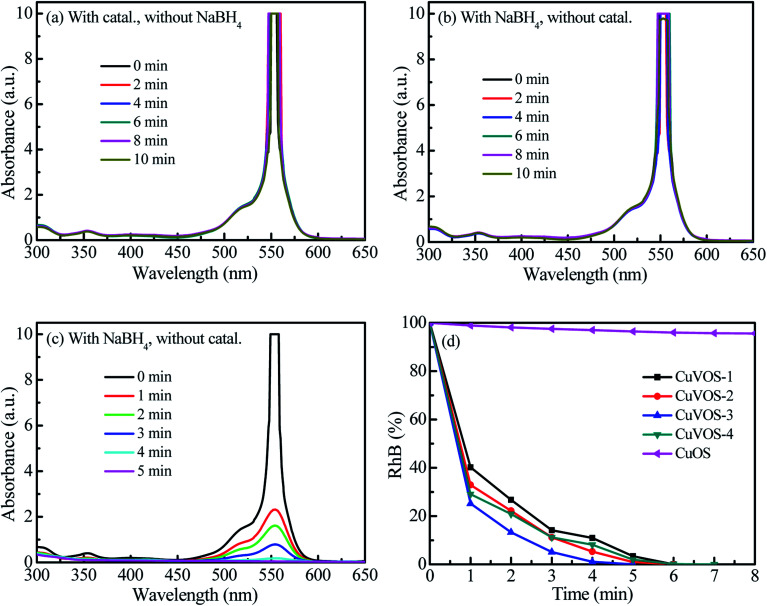
(a) Reduction of RhB with Cu*V*OS-3 catalyst without NaBH_4_, (b) reduction of RhB by NaBH_4_ without catalyst, (c) reduction of RhB by Cu*V*OS-3 catalyst with NaBH_4_, and (d) reduction of RhB by Cu*V*OS catalyst prepared at different N_2_H_4_ contents with NaBH_4_ under dark.

In order to evaluate the reduction performance of the Cu*V*OS catalyst, the catalysts were also used to reduce MB and RhB, and the results were shown in Fig. 10 and 11. The control experiments of Cu*V*OS catalysts for MB and RhB reduction are shown in Fig. 10a, b and 11a, b, respectively. Moreover, Fig. 10c and 11c indicate the reduction of MB and RhB dyes, respectively, by Cu*V*OS-3 catalysts. The strongest peaks of MB at 560–700 nm was completely degraded in the presence of Cu*V*OS-3 catalyst in 8 min, while CuOS degraded only less than 10% (Fig. 10d). The pseudo first-order apparent reaction rate constant (*k*_app_) of MB reduction over Cu*V*OS is as follows: Cu*V*OS-3 (*k*_app_ = 0.72 min^−1^) > Cu*V*OS-4 (*k*_app_ = 0.59 min^−1^) > Cu*V*OS-2 (*k*_app_ = 0.48 min^−1^) > Cu*V*OS-1 (*k*_app_ = 0.29 min^−1^) > CuOS (*k*_app_ = 0.01 min^−1^). Similar to MB, the pseudo first-order apparent reaction rate constant (*k*_app_) of RhB reduction over Cu*V*OS is also calculated as follows: Cu*V*OS-3 (*k*_app_ = 0.93 min^−1^) > Cu*V*OS-2 (*k*_app_ = 0.78 min^−1^) > Cu*V*OS-4 (*k*_app_ = 0.74 min^−1^) > Cu*V*OS-1 (*k*_app_ = 0.51 min^−1^) > CuOS (*k*_app_ = 0.004 min^−1^). Based on the above analyses, the reduction capability of Cu*V*OS on pollutants is as follow: MO > RhB > MB. It is further demonstrated that the bimetallic oxysulfide Cu*V*OS catalyst has great prospects in industrial applications for wastewater treatment.

### 3.9 The proposed reduction reaction mechanism

**Fig. 12:**
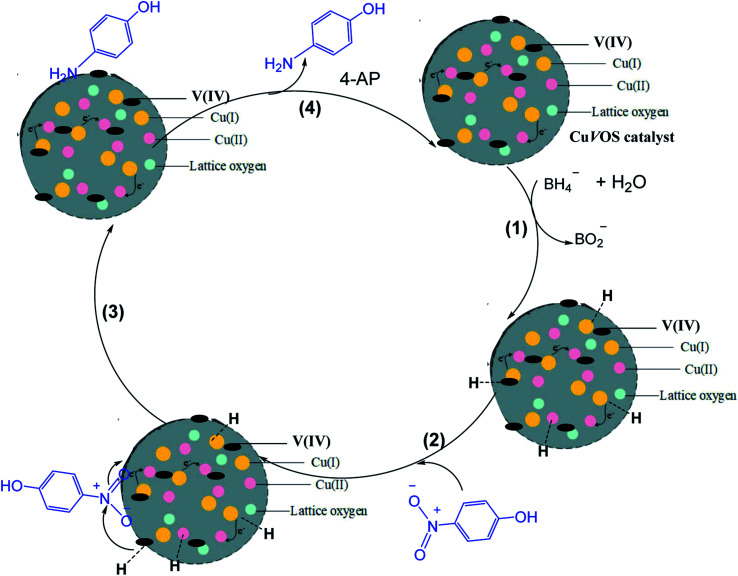
The schematic reaction of 4-NP in presence of the Cu*V*OS catalyst.

Fig. 12 shows a possible reaction mechanism for the catalytic reduction of 4-NP by Cu*V*OS catalyst in the presence of NaBH_4_ as a reducing agent. When NaBH_4_ is dissociated, the BH_4_^−^ ion is released and attached on the surface of the catalyst (step 1). Then, the hydride ion is bonded covalently with the Cu*V*OS (step 2). Simultaneously, the adsorption of nitro groups of 4-NP, will took place on the surface of the catalyst as it is shown in step 3. The strong interactions between adsorbed 4-NP and covalently bonded hydrogen atoms will occur. Then, the adsorbed nitro groups will react with the hydride ion and reduction took place through the transfer of electron from donor BH_4_^−^ to the acceptor 4-NP and lead to desorption of 4-AP product.^49^ In addition, Cu(i) and Cu(ii) in the Cu*V*OS catalyst can transfer the electron and to accelerate the reduction reaction.^50,51^ Moreover, the V(iv) atom will also accept the electrons and transfer for the reduction purpose. Fig. 12 shows the mechanism of 4-NP reduction catalyzed by Cu*V*OS.

## 4. Conclusions

A copper–vanadium bimetallic oxysulfide Cu*V*OS catalyst was successfully synthesized by facile method and had excellent reducing activities for 4-NP, MO, MB, and RhB. It showed that, a 100 mL of 4-NP (20 ppm) solution was completely reduced in the presence of 5 mg of Cu*V*OS-3. Moreover, the complete reduction of 100 mL of MO, RhB, and MB solutions (100 ppm) were also achieved within 2 min, 6 min, and 5 min, respectively, with 5 mg Cu*V*OS-3. The stability of Cu*V*OS-3 was relatively good after using repeatedly for the reduction of 4-NP. Hence, the Cu*V*OS prepared in the presence of optimum amount of N_2_H_4_ is an efficient catalyst for reducing 4-NP and other organic dyes and may have a great potential for industrial application.

## Conflicts of interest

There are no conflicts to declare.

## Supplementary Material
